# Globalization and vulnerable populations in times of a pandemic: a Mayan perspective

**DOI:** 10.1186/s13010-020-00097-0

**Published:** 2020-12-14

**Authors:** Claudia Sotomayor, Alejandra Barrero-Castillero

**Affiliations:** 1 Pellegrino Center for Clinical Bioethics, Georgetown University, Washington DC, USA; 2 Department of Neonatology, Beth Israel Deaconess Medical Center, Boston, MA USA; 3 Department of Pediatrics, Harvard Medical School, Boston, MA USA

Globalization has a double face that brings to the table free flow of trade, investments, and profits across nations with the hope of improving global integration that eventually will produce the best economic, social, and political outcomes for humanity. From a public health perspective, globalization has improved health and life expectancy in many populations, but unfortunately, it has endangered many others due to the erosion of the environment, the global division of labor, the exacerbation of the rich-poor gap between and within countries, and the accelerating spread of consumerism [1].

As a result, global health conditions are marked by inequities due mostly to poverty and lack of access to healthcare services. In a Pandemic setting, these disparities are exacerbated. Experience from the influenza pandemic in 2009, show that there are several causes for such event [2, 3]. First, low-income and minority workers may have difficulty in adhering to directives to stay home from work because of the nature of their jobs as part of the essential workforce and the necessity to work [4]. Further, their living conditions may be marked by overcrowding and sometimes lack of basic sanitation as well as psychosocial stressors and conditions that may increase the risk and susceptibility to develop or worsen underlying medical conditions [5, 6].

Indigenous populations around the world face even more dramatic challenges, experiencing a higher degree of socio-economic disadvantage, marginalization, poor access to health care and essential services, and lack of access to effective monitoring and early-warning systems. Even in the instances where health care services can be reached, indigenous communities frequently face discrimination, disproportionally increasing the risk and vulnerability in public health emergencies like during this global unprecedented pandemic [2].

## The Mayan experience

A good example of how globalization affects indigenous and vulnerable populations is the Mayan Communities in the Yucatan Peninsula. In our early years of practicing physicians, we volunteered at a non-profit organization that provided community health care to Mayan Communities in the State of Quintana Roo, Mexico. The experience not only shaped our future career choices, but taught us the complexity that entails providing adequate healthcare to marginalized and vulnerable populations. We witnessed a rooted distrust of the healthcare system, the corruption of the government, a high degree of socio-economic marginalization, and many underlying host factors and medical conditions that increase the risk of diseases and complications, many of which we were unable to subside. These remote communities are often immersed in the jungle with low infrastructure, difficult road conditions, poor sanitation, and no or minimal access to health and social services.

Due to the financial constraints, many people from the communities go to larger cities -such as Cancun- to work in the hotel industry. Before the coronavirus-era tourism was thriving, cruise ships were coming and going to and from the Riviera Maya, spring breakers were enjoying the beaches, people all around the world traveled to witness the natural beauty of this land. Unfortunately, along with their contribution to the economy, this year tourists also brought SARS-COV-2 with them. The first three confirmed cases in the area were announced on March 10th, and it was until March the 30th when social distancing measures were issued, hotels were closed and people had to go back to their hometowns. And so, they took the virus with them. To support the population, Mexican government has issued messages via radio and brochures and guides translated appropriately in multiple native languages, including Mayan. In terms of healthcare access; small community clinics frequently under-stocked with intermittent and limited medical staff, have been even more susceptible to workforce and material shortages due to the fact that medical personnel are recruited to assist in front lines at local hospitals.

Another major problem- often forgotten in a globalized world- is the cultural context. Some of these communities are still very rooted in their Mayan traditions that sometimes clash with the western view of medicine and healing. Understanding the interpretation of illness, health and healthcare of the communities is crucial when setting up preparedness plans. Thoughtful consideration of the community context helps develop a line of communication that is appropriate for them and avoids clashing cultures. In the case of the Mayas, it is important to note that life, illness and health are interrelated events, and they have a direct relation with their gods and their ancestors. Life is interrelated with the physical world and the gods from the sky, earth, and underworld. This interconnectedness is reflected in the Mayan view of illness [7]. When it comes to healthcare choices, Mayans have a communitarian approach where a decision is not taken autonomously by one individual, but rather as a communal decision where the extended family and the H-men (Mayan spiritual healer) participate [8]. Mental reasoning is not taken into consideration when making healthcare choices, because it is believed that the human heart is the receptor of the divine essence that comes from the “Heart of the Sky” and the “Heart of the Earth”, therefore it is only the heart that enables people to use their good sense and not the brain [9].

## Future directions focused on advocating and protecting indigenous and vulnerable communities

With this background one can imagine that imposing social distancing practices due to a novel virus can be difficult if not addressed in a culturally sensitive manner. It is well known that pandemic preparedness and response must occur within a social, cultural, and historical context of preexisting health disparities [2]. In this case, understanding their views on health, illness and healthcare and including the H-man in the planning and implementation process of prevention and mitigation strategies is crucial to gain the trust of the community. Building bridges of communication and trust between the leaders of our native communities is key to be able to protect these communities and to improve their adherence to societal guidelines. However, in a crisis there is no time to build those bridges.

With this unfortunate event, one thing is clear, globalization has unintended health risks, and marginalized communities are left in an even more vulnerable position. It is time to call the global community to work together to fight the pandemic. This call is based on the notion of social responsibility in health care - the moral duty held by all societies to promote health, prevent and treat diseases, and to provide the highest attainable standard of health. Article 14 of the UNESCO [10] Universal Declaration on Bioethics and Human Rights, presents the principle of social responsibility and health in the field of bioethics “to ensure, whenever possible, that progress in science and technology contributes to justice, equity and to the interest of humanity”. The stakeholders are numerous and include governments, groups of people organized within societies, commercial companies, political organizations, educational institutions, and others. Now 5 months into the pandemic, even when it is still recommended to avoid non- essential travel and social distancing, the tourist area of Quintana Roo has started to reopen their hotels and beaches and have welcomed travelers around the world with a reduced occupancy of 30%. While slowly reopening the economy is necessary and beneficial in our globalized world, being mindful on how to reopen, provide protection, support and equitable access to health care services to vulnerable populations like our indigenous communities around the world in a culturally competent manner is imperative.
